# Thyroid Function Abnormalities in COVID-19 Patients

**DOI:** 10.3389/fendo.2020.623792

**Published:** 2021-02-19

**Authors:** Weibin Wang, Xingyun Su, Yongfeng Ding, Weina Fan, Weibin Zhou, Junwei Su, Zhendong Chen, Hong Zhao, Kaijin Xu, Qin Ni, Xiaowei Xu, Yunqing Qiu, Lisong Teng

**Affiliations:** ^1^Department of Surgical Oncology, The First Affiliated Hospital, Zhejiang University School of Medicine, Hangzhou, China; ^2^Department of Medical Oncology, The First Affiliated Hospital, Zhejiang University School of Medicine, Hangzhou, China; ^3^Department of Intensive Care Unit (ICU), The First Affiliated Hospital, Zhejiang University School of Medicine, Hangzhou, China; ^4^Department of Endocrinology, The First Affiliated Hospital, Zhejiang University School of Medicine, Hangzhou, China; ^5^State Key Laboratory for Diagnosis and Treatment of Infectious Diseases, National Clinical Research Center for Infectious Diseases, Collaborative Innovation Center for Diagnosis and Treatment of Infectious Diseases, Department of Infectious Diseases, The First Affiliated Hospital, College of Medicine, Zhejiang University, Hangzhou, China

**Keywords:** COVID-19, thyroid function, abnormality, thyroid stimulating hormone, pathogenesis

## Abstract

**Purpose:**

The novel coronavirus COVID-19, has caused a worldwide pandemic, impairing several human organs and systems. Whether COVID-19 affects human thyroid function remains unknown.

**Methods:**

Eighty-four hospitalized COVID-19 patients in the First Affiliated Hospital, Zhejiang University School of Medicine (Hangzhou, China) were retrospectively enrolled in this study, among which 22 cases had complete records of thyroid hormones. In addition, 91 other patients with pneumonia and 807 healthy subjects were included as controls.

**Results:**

We found that levels of total triiodothyronine (TT3) and thyroid stimulating hormone (TSH) were lower in COVID-19 patients than healthy group (p < 0.001). Besides, TSH level in COVID-19 patients was obviously lower than non-COVID-19 patients (p < 0.001). Within the group of COVID-19, 61.9% (52/84) patients presented with thyroid function abnormalities and the proportion of thyroid dysfunction was higher in severe cases than mild/moderate cases (74.6 vs. 23.8%, p < 0.001). Patients with thyroid dysfunction tended to have longer viral nucleic acid cleaning time (14.1 ± 9.4 vs. 10.6 ± 8.3 days, p = 0.088). To note, thyroid dysfunction was also associated with decreased lymphocytes (p < 0.001) and increased CRP (p = 0.002). The correlation between TT3 and TSH level seemed to be positive rather than negative in the early stage, and gradually turned to be negatively related over time.

**Conclusion:**

Thyroid function abnormalities are common in COVID-19 patients, especially in severe cases. This might be partially explained by nonthyroidal illness syndrome.

## Introduction

The outbreak of coronavirus disease 2019 (COVID-19), caused by severe acute respiratory syndrome coronavirus 2 (SARS-CoV-2), has rapidly spread worldwide and led to the declaration of Public Health Emergency of International Concern by the World Health Organization (WHO) (1, 2). As of June 14, 2020, a total of 7,844,978 cases have been confirmed worldwide, among them, 428,045 people have died of COVID-19. Patients infected with COVID-19 display mainly symptoms similar with pneumonia such as fever, fatigue, cough, shortness of breath (3, 4). And many patients have symptoms outside of the respiratory system including poor appetite, diarrhea, nausea, vomiting and palpitation (3, 4). The main management of COVID-19 infection is supportive, and acute respiratory distress syndrome (ARDS) induced respiratory failure is the leading cause of mortality (3, 5–7).

Severe and complex effects on several human organs and systems including respiratory, immune, digestive, circulatory, hepatic, renal, and hematological systems have been reported in COVID-19 patients (3–6). Whether COVID-19 affects human thyroid function remains unknown. Previously, thyroid dysfunction was identified in patients with severe acute respiratory syndrome (SARS) caused by a different strain of coronavirus (8). Therefore, COVID-19 may also influence the function of thyroid. Recently, Brancatella et al. reported the first case of subacute thyroiditis after SARS-CoV-2 infection (9). Both reports therefore indicate that the thyroid gland may also be a target organ of SARS-CoV-2. Investigating thyroid function in COVID-19 patients might help to uncover the pathogenesis of SARS-CoV-2 and provide effective information for clinical practice.

In the present study, 84 hospitalized COVID-19 patients were enrolled retrospectively from the First Affiliated Hospital, Zhejiang University School of Medicine (Hangzhou, China). In addition, 91 other patients with pneumonia and 807 healthy subjects were included as controls. The thyroid function in COVID-19 was compared with that in pneumonia patients, and its relationship with disease severity, viral nucleic acid cleaning time, auto-antibodies, leukocytes, inflammatory biomarkers and cytokines was also investigated. Furthermore, the nature history of thyroid function during patients’ recovery were also studied to depict the development of thyroid dysfunction causing by SARS-CoV-2.

## Materials and Methods

### Participants

Ninety-six hospitalized patients from the First Affiliated Hospital, Zhejiang University School of Medicine (Hangzhou, China), who were definitively diagnosed as COVID-19 according to WHO interim guidance (10), participated. They represented all COVID-19 patients admitted to our hospital in the period from January 22nd to March 16th, 2020, and diagnosed by the positive of nucleic acid of SARS-CoV-2 *via* nasal pharyngeal swab or phlegm. Within two days of admission, 85 of the 96 patients were assessed for thyroid function. One patient was excluded due to pregnancy leaving a total of 84 patients enrolled. In addition, 91 non-COVID-19 pneumonia patients from the Department of Respiratory Diseases or the ICU who were infected by bacteria, fungus, and virus, and 807 healthy subjects who underwent annual routine physical checkup in our Health Management Center were included as controls. Since the examination of thyroid function is included in the panel of annual routine physical checkup, the thyroid hormone levels of these healthy subjects are available. Non-COVID-19 pneumonia cases were matched for age, gender, and disease severity with COVID-19 patients.

Clinical classification of COVID-19 was based on the Handbook of COVID-19 Prevention and Treatment (11), and cases were judged as severe if they met any of the following criteria: 1) respiratory rate over 30 breaths/min; 2) oxygen saturation ≤93% at a rest state; and 3) arterial partial pressure of oxygen (PaO2)/oxygen concentration (FiO2) ≤300 mmHg. In additions, patients with >50% lesions progression within 24 to 48 h in lung imaging were also regarded as severe cases. Patients who presented with classical respiratory tract symptoms but did not reach the criteria of “Severe” were classified as “Moderate” while those with no or mild symptoms without CT pneumonia manifestation were classified as “Mild”. All cases provided informed consent. The research was approved by the Ethics Committee of the First Affiliated Hospital, Zhejiang University School of Medicine.

### Data Collection

Thyroid hormones including triiodothyronine (TT3), thyroxine (TT4), and thyroid stimulating hormone (TSH) were successfully collected in all studied groups. In COVID-19 patients, we also have 22 patients with complete records of thyroid hormones including TT3, TT4, free triiodothyronine (fT3), free thyroxine (fT4) and TSH. All COVID-19 patients had record of TT3, TT4, and TSH at admission. We also collected the clinicopathologic characteristics of age, gender, disease severity, and lab data of inflammatory biomarker (leukocytes, C-reactive protein, procalcitonin), inflammatory cytokines (interleukin-6, interleukin-10, tumor necrosis factor-α, interferon-γ), auto-antibodies (thyroglobulin antibody, thyroid peroxidase antibody), and viral nucleic acid cleaning time of patients. The viral nucleic acid cleaning time was defined as the period from diagnosed as positive to negative of nucleic acid of SARS-CoV-2 *via* nasal pharyngeal swab or phlegm, and the negative result was repeated twice with interval of one day.

### Research Procedures and Statistical Analysis

Between COVID-19 patients with complete and incomplete records of thyroid hormones, no statistic difference was found in rate of thyroid dysfunction, disease severity or viral nucleic acid cleaning time (**Table S1**). Then we compared age, gender, and thyroid function among COVID-19, non-COVID-19 pneumonia patients and healthy subjects. The associations between thyroid function and disease severity, inflammatory biomarker (leukocytes, C-reactive protein, procalcitonin), inflammatory cytokines (interleukin-6, interleukin-10, tumor necrosis factor-α, interferon-γ), auto-antibodies (thyroglobulin antibody, thyroid peroxidase antibody), and viral nucleic acid cleaning time were analyzed. We also analyzed the dynamic changes of thyroid function during patients’ recovery period.

Statistical analysis was conducted by SPSS (version 21.0) (SPSS Inc., Chicago, IL, USA) and R language (Version 3.6.3). Pearson chi-square test and analysis of variance (ANOVA) were used to analyze the characteristics and thyroid function among COVID-19, non-COVID-19 pneumonia patients and healthy subjects (False Discovery Rate (FDR) correction was used for multiple comparison), and to establish the factors associated with dysfunction of thyroid. Pearson correlation was performed in the correlation analysis. In addition, a polynomial regression curve was fitted between time after hospitalization and TSH or TT3 levels. For all analyses, p <0.05 was regarded as statistically significant.

## Results

### Characteristics of Participants

The COVID-19 patients had a mean age of 57.3 ± 14.5 years old and 63% (53/84) were male. They did not differ statistically from non-COVID-19 pneumonia patients or healthy subjects in age or gender (**Table 1**). Since the COVID-19 group and non-COVID-19 pneumonia group were matched on disease severity, we didn’t find any difference on clinical classification between the two groups (**Table 1**). Patients having thyroid dysfunction were those with any abnormality in TT3, TT4, or TSH. Based on quantification of thyroid hormones, a total of 52 (52/84, 61.9%) COVID-19 patients have thyroid dysfunction. Among thyroid dysfunction cases, two patients had decreased TSH accompanied by slightly enhanced TT4 while the remained 50 patients presented with lower levels of TT4, TT3, or TSH (**Figure S1**).

**Table 1:** Comparison of clinical features among COVID-19, non-COVID-19 pneumonia patients and healthy subjects.

Characteristics	COVID-19 patients(N = 84)	Non-COVID-19 pneumonia patients(N = 91)	Healthy subjects(N = 807)	*p* value			
	Mean ± SD or n (%)	Mean ± SD or n (%)	Mean ± SD or n (%)	COVID-19 vs. Non-COVID-19	COVID-19 vs. Healthy subjects		
Mean age (yrs)	57.3 ± 14.5	60.1 ± 16.7	57.7 ± 13.0	0.472	0.782		
Gender							
Male	53 (63.1%)	60 (65.9%)	474 (58.7%)	0.695	0.695		
Female	31 (36.9%)	31 (34.1%)	333 (41.3%)				
Clinical classifications							
Mild and moderate	21 (25.0%)	24 (26.4%)	–	0.835	–		
Severe and critical	63 (75.0%)	67 (73.6%)	–		–		
Thyroid Function							
TT4 (nmol/L)	99.04 ± 25.96	82.28 ± 26.47	97.25 ± 17.13	***0.000***	0.391		
TT3 (nmol/L)	1.02 ± 0.32	0.92 ± 0.38	1.59 ± 0.24	0.059	***0.000***		
TSH (mIU/L)	0.62 ± 0.62	1.07 ± 0.94	1.55 ± 0.94	***0.001***	***0.000***		

TT4, total thyroxine or tetraiodothyronine, normal range 62.68–150.84 nmol/L; TT3, total triiodothyronine normal range 0.89–2.44 nmol/L; TSH, thyroid-stimulating hormone, normal range 0.35–4.94 nmol/L.

p values were False Discovery Rate (FDR)-corrected. p value in bold was regarded as statistically significant.

### Thyroid Dysfunction in COVID-19 Patients

**Figure 1:**
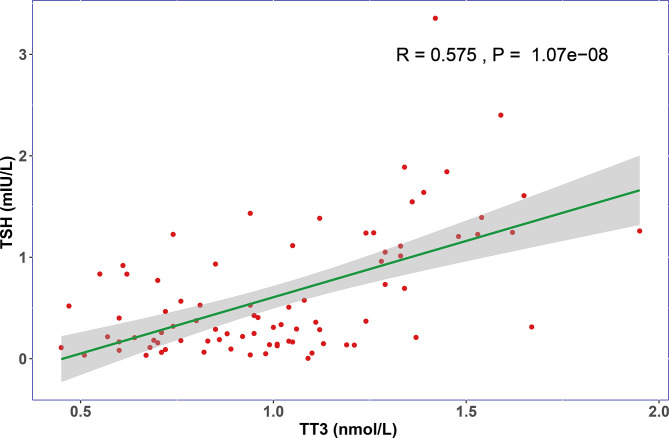
The relationship of TT3 and TSH levels on admission. The TT3 and TSH levels in COVID-19 patients are positively correlated (R = 0.575, P < 0.001).

When compared with healthy subjects, the levels of TT3 and TSH were significantly lower in COVID-19 patients (p < 0.001), while no significant difference was found in TT4 (p = 0.391) (**Table 1**). We then focused our research on TT3 and TSH. Next, we investigated the thyroid function alterations between COVID-19 and non-COVID-19 pneumonia patients. The level of TT3 in COVID-19 patients (1.02 ± 0.32 nmol/L) was similar with that in non-COVID-19 patients (0.92 ± 0.38 nmol/L) (p = 0.59) (**Table 1**). However, the TSH level was significantly lower in COVID-19 cases (0.62 ± 0.62 mIU/L vs. 1.07 ± 0.94 mIU/L, p < 0.001) (**Table 1**). TSH secreted by adenohypophysis normally drives the output of thyroid hormones and will be inhibited by enhanced thyroid hormones (particularly fT3) in terms of the negative feedback loop of pituitary-thyroid axis. However, our correlation analysis revealed that the levels of TT3 and TSH were positively correlated, instead of negatively related, in COVID-19 patients (R = 0.575, p < 0.001) (**Figure 1**). Correlation analysis showed that fT3 was in line with TT3 (p < 0.001) (**Figure S2**).

### Analysis of Clinical Values for Thyroid Dysfunction in COVID-19 Patients

COVID-19 patients were further divided into a thyroid dysfunction subgroup and a normal subgroup according to TT3, TT4, and TSH levels. No obvious difference was found in age and sex between these two subgroups (**Table 2**). Abnormal thyroid function was more commonly detected in severe cases than mild/moderate cases (74.6 vs. 23.8%, p < 0.001) (**Table 2**). Interestingly, thyroid dysfunction tended to be associated with longer viral nucleic acid cleaning time (14.13 ± 9.39 vs. 10.56 ± 8.29 days, p = 0.088). Additionally, we identified increased levels of leucocytes (p < 0.001), neutrophils (p < 0.001), CRP (p = 0.002) and PCT (p = 0.054), and decreased level of lymphocytes (p < 0.001) in thyroid dysfunction group. Meanwhile, we did not find any significance in levels of auto-antibodies (thyroglobulin antibody, thyroid peroxidase antibody) and cytokines (IL-6, IL-10, TNF-α, IFN-γ) (**Table 2**).

**Table 2:** Clinical characteristics and selected laboratory abnormalities of COVID-19 patients with and without thyroid dysfunction.

	Thyroid dysfunction* (N = 52) Mean ± SD or n (%)	Normal (N = 32) Mean ± SD or n (%)	P value
Gender			
Male	35 (66.0%)	18 (34.0%)	0.308
Female	17 (54.8%)	14 (45.2%)	
Clinical classifications on admission			
Mild and moderate	5 (23.8%)	16 (76.2%)	***0.000***
Severe and critical	47 (74.6%)	16 (25.4%)	
Viral nucleic acid cleaning time (days)	14.1 ± 9.4	10.6 ± 8.3	0.088
Thyroid auto-antibodies			
TPOAb (IU/ml, normal range 0–5.61)	23.95 ± 38.12	24.71 ± 57.08	0.945
TGAb (IU/ml, normal range 0–4.11)	35.05 ± 142.01	20.98 ± 47.31	0.613
Cytokines			
IL-6 (pg/ml; normal range 0–6.61)	59.27 ± 98.24	51.99 ± 95.17	0.748
IL-10 (pg/ml; normal range 0–2.31)	8.15 ± 10.92	6.11 ± 7.58	0.378
TNF-α (pg/ml; normal range 0–33.27)	69.14 ± 259.33	31.27 ± 38.23	0.438
IFN-γ (pg/ml; normal range 0–20.06)	32.92 ± 73.10	30.92 ± 47.39	0.895
Blood routine tests			
Leucocytes (x10^9^/L; normal range 4–10)	8.71 ± 5.33	4.92 ± 1.64	***0.000***
Neutrophils (x10^9^/L; normal range 2–7)	7.97 ± 5.30	3.40 ± 1.51	***0.000***
Lymphocytes (x10^9^/L; normal range 0.8–4)	0.62 ± 0.33	1.09 ± 0.41	***0.000***
Platelets (x10^9^/L; normal range 83–303)	189.13 ± 71.13	209.38 ± 75.93	0.221
Haemoglobin (g/L; normal range: male 131–172, female 113–151)	132.50 ± 17.14	137.16 ± 15.69	0.216
Infection-related biomarkers			
Procalcitonin (ng/L; normal range 0–0.5)	0.22 ± 0.46	0.06 ± 0.45	0.054
C reactive protein (mg/ml; normal range 0–8)	38.14 ± 37.01	15.60 ± 17.73	***0.002***
Blood biochemistry			
Globulin (g/L; normal range 20–40)	29.34 ± 5.85	27.82 ± 4.16	0.204

TPOAb, thyroid peroxidase antibody; TGAb, thyroglobulin antibody; IL-6, interleukin-6; IL-10, interleukin-10; TNF-α, tumor necrosis factor α; IFN-γ, interferon-γ.

p value in bold was regarded as statistically significant.

*Thyroid dysfunction indicates any abnormalities in the levels of TT4, TT3, or TSH.

### The Natural History of Thyroid Dysfunction in COVID-19 Patients

**Figure 2:**
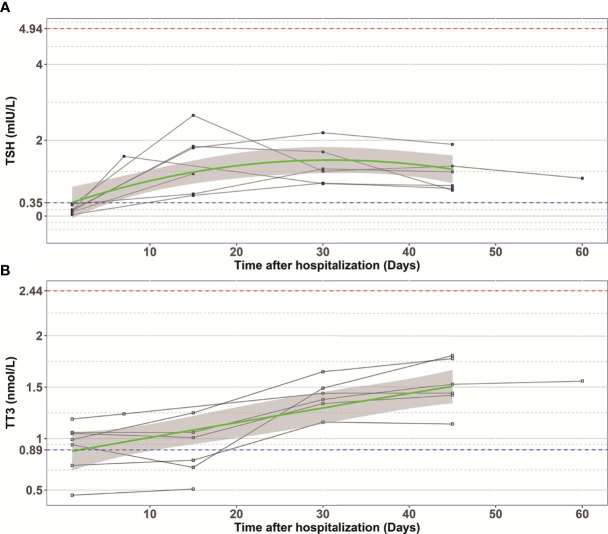
The changes of TSH **(A)** and TT3 **(B)** levels during hospitalization in COVID-19 patients with abnormal TSH level on admission. Every polyline represents the variation trend of TSH or TT3 level of one patient. Dashed blue lines, the lower limit of normal TSH (0.35 mIU/L) and TT3 (0.89 nmol/L) value; Dashed red lines, the upper limit of normal TSH (4.94 mIU/L) and TT3 (2.44 nmol/L) value; Green curves represent the fitting of data; Grey shaded areas, depict the 95% confidence band for the fitted curve.

**Figure 3:**
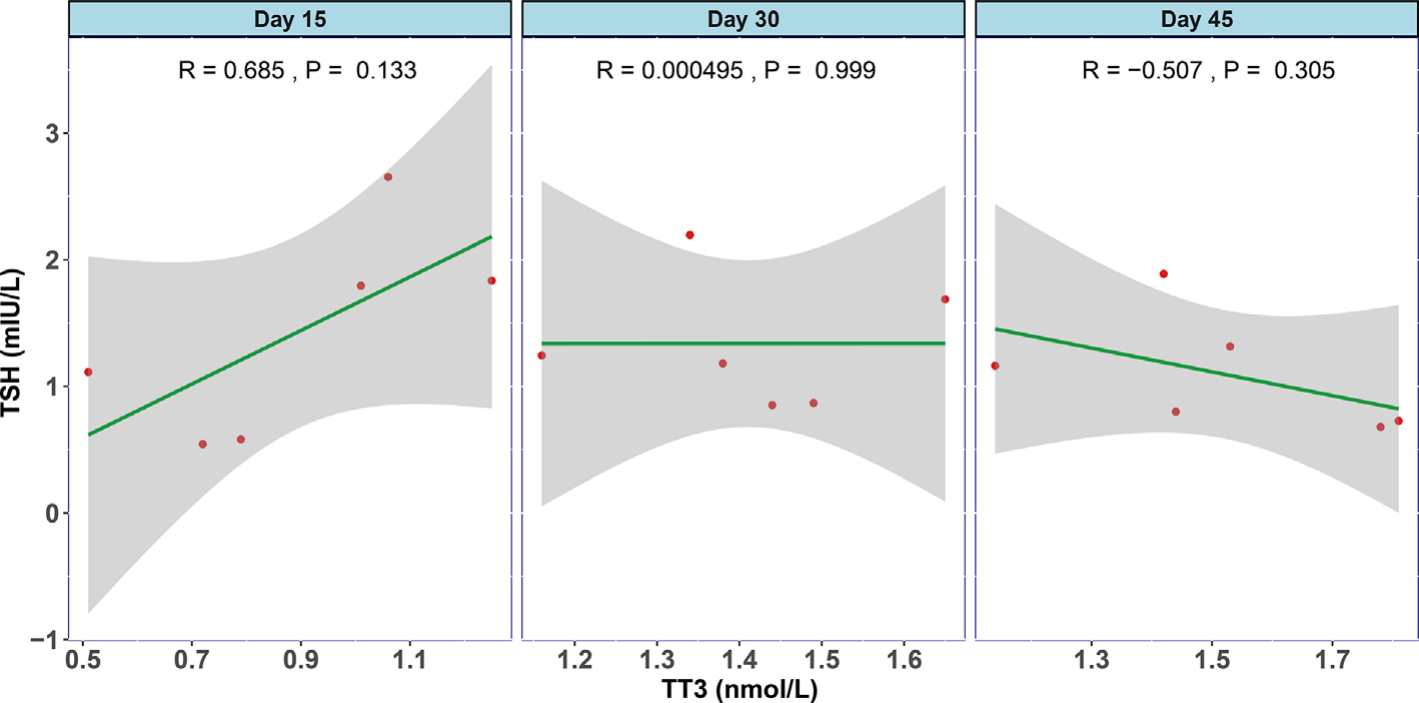
The association between TT3 and TSH levels in different disease stages of COVID-19. The levels of TT3 and TSH tend to be positively correlated in the early stage (Day 15) and turn towards negatively correlated in Day 45, but the p values are not significant.

In order to examine the natural history of thyroid dysfunction induced by SARS-CoV-2, we analyzed seven patients with records of thyroid function during their recovery period. All seven patients had lower than normal range of TSH levels on admission, and none of them was treated by glucocorticoid or thyroxine. We observed that the levels of TT3 and TSH increased gradually within 2 months after hospitalization (**Figure 2**). At Day 30, all the patients’ TT3 and TSH levels recovered to normal without any thyroid hormone replacement (**Figure 2**). We further found the correlation between TT3 and TSH levels seem to shift from a positive pattern to a negative pattern overtime, indicating a recovery of the pituitary-thyroid axis. However, given limited patient number, the p-values did not reach statistical significance (**Figure 3**).

## Discussion

COVID-19 is an infectious illness that has caused a pandemic worldwide. As a novel type of disease with high infectivity and mortality, the pathophysiology of COVID-19 has not been fully studied. A number of studies have reported severe and complex effects of COVID-19 in several human organs and systems including respiratory, immune, digestive, circulatory, hepatic, renal, and hematological systems (6). However, whether COVID-19 affects human thyroid function remains unknown. Here, we report the influence of COVID-19 on thyroid function. We found that COVID-19 patients presented with lower levels of TT3 and TSH than healthy subjects while their TSH levels were considerably lower than non-COVID-19 pneumonia patients. We also observed that thyroid dysfunction in COVID-19 patients may recover without thyroid hormone replacement within 30 days. This seems to mimic the pattern seen in patients with non-thyroidal illness (NTI).

Nonthyroidal illness syndrome presents as abnormal thyroid function in serious diseases other than thyroid disorders, including infection, cancer, cardiovascular and gastrointestinal disease, burn, and trauma (12). It is well established that NTI is a consequence of an acute phase response to severe systemic illness or macronutrient restriction and usually presents as decreased plasma T3 level, or low or normal T4 and TSH levels (12, 13). The phenomenon of decreased T3 and TSH in COVID-19 patients was consistent with NTI. In COVID-19 patients, a profile of cytokines, such as IL-2, IL-6, IL-7, INF-γ, and TNF-α, is associated with disease severity and mortality of patients (3, 5, 7, 14). Our results also showed that thyroid dysfunction was associated with increased inflammation biomarkers including CRP and leucocytes, indicating inflammatory reaction played an important role in thyroid dysfunction of COVID-19. Therefore, serious infection in COVID-19 is a primary cause of NTI.

Furthermore, even though the disease severity was matched, we still found the TSH level of COVID-19 patients was significantly lower than that in non-COVID-19 pneumonia patients. This suggests thyroid function abnormalities in COVID-19 patients cannot be totally explained by NTI, possibly because of the attack of SARS CoV-2 virus. The wide distribution of COVID-19 nucleic acid in respiratory tract, saliva, feces, and breastmilk indicates that direct viral attack to the target cells may be an alternative reason (15–17). Angiotensin-converting enzyme 2 (ACE2) is a receptor providing the main entry site for SARS-CoV to invade human cells, and this in turn facilitates direct damage of virus through the course of infection (18, 19). Li et al. recently reported that ACE2 was highly expressed in the thyroid (20), suggesting that the thyroid gland may be a potential target for direct attack of COVID-19. Our study showed that thyroid dysfunction tended to be associated with viral nucleic acid cleaning time, indicating virus infection and replication may account for the abnormal thyroid hormones. However, our study also showed that disease severity, which may influence the viral nucleic acid cleaning time, was associated with thyroid dysfunction, thus the true relationship of thyroid function and viral nucleic acid cleaning time need to be further studied.

In patients with SARS caused by another strain of coronavirus (8), severe pathologic injury in follicular epithelial cells with follicular distortion and collapse was found in thyroid glands (21). After investigating the endocrine cells in the pituitary gland of five SARS patients, Wei et al. found that TSH positive cells were significantly decreased (22), indicating thyroid epithelial cells, as well as endocrine cells of adenohypophysis, may be attacked and damaged by coronavirus. Thus, we speculated that COVID-19 may have similar pathogenesis as SARS, explaining why the TSH level in COVID-19 patients was significantly lower than non-COVID-19 patients. In the present study, we also noticed 7 patients, who had lower than normal levels of TSH and TT3 on admission, with normalization by Day 30. Furthermore, the malfunctional feedback between TT3 and TSH returned to work overtime, indicating a recovery of the pituitary-thyroid axis abnormalities as well. A recent case report of thyroiditis after SARS-CoV-2 infection came up by Brancatella et al. confirmed this hypothesis. That case displayed thyroid dysfunction followed by a triphasic course including thyrotoxicosis, hypothyroidism, and euthyroidism, and then recovered to normal in one month (9).

There are several limitations which might cause potential bias. The study is single centered, with limited sample size, which may lead to bias of the study. Also, the study was conducted retrospectively with little attention paid to thyroid function during treatment of COVID-19, most patients had not dynamically monitored the thyroid function. Furthermore, only 22 patients had complete thyroid function including fT3 and fT4. Also, COVID-19 patients were admitted to hospital at different disease stages with different severity, and most patients with mild symptom did not have thyroid function tests, which may also cause bias to an extent. Thus, more patients from multiple centers should be analyzed, and complete record and dynamic changes of thyroid function should be concerned and investigated prospectively in the future studies.

In conclusion, the current study demonstrated that thyroid function abnormalities were common in COVID-19 patients, especially in severe cases. The thyroid dysfunction seems to dynamically change within the course of disease and recover gradually and spontaneously. While this may be partially explained by non-thyroidal illness syndrome, it is also possible that the thyroid gland is a direct target of the SARS CoV-2 virus.

## Data Availability Statement

The original contributions presented in the study are included in the article/**Supplementary Material**. Further inquiries can be directed to the corresponding authors.

## Ethics Statement

The studies involving human participants were reviewed and approved by Ethics Committee of the First Affiliated Hospital, Zhejiang University School of Medicine. The patients/participants provided their written informed consent to participate in this study.

## Author Contributions

WW, XS, YD, and WF designed the study, analyzed the data and wrote the paper. JS, ZC, HZ, KX, QN, and XX collected data and performed the study. LT and YQ designed the study, supervised the whole process, and critically revised the manuscript. All authors contributed to the article and approved the submitted version.

## Funding

This study was supported by Zhejiang Provincial Science and technology department key R & D plan emergency project (No. 2020c03123-8).

## Conflict of Interest

The authors declare that the research was conducted in the absence of any commercial or financial relationships that could be construed as a potential conflict of interest.

## Supplementary Material

Supplementary Figure 1 The distribution of thyroid hormones abnormalities in COVID-19 patient.

Supplementary Figure 2 The respective association between fT3 and TT3 levels in COVID-19 patients on admission. Correlation analysis shows that fT3 is positively related with TT3 (R = 0.668, P < 0.01).
