# Supplementary figures and images for: Thyroid Function Abnormalities in COVID-19 Patients
Source: Front Endocrinol (Lausanne). 2021 Feb 19;11:623792. doi: 10.3389/fendo.2020.623792 (PMC7933556; doi:10.3389/fendo.2020.623792)

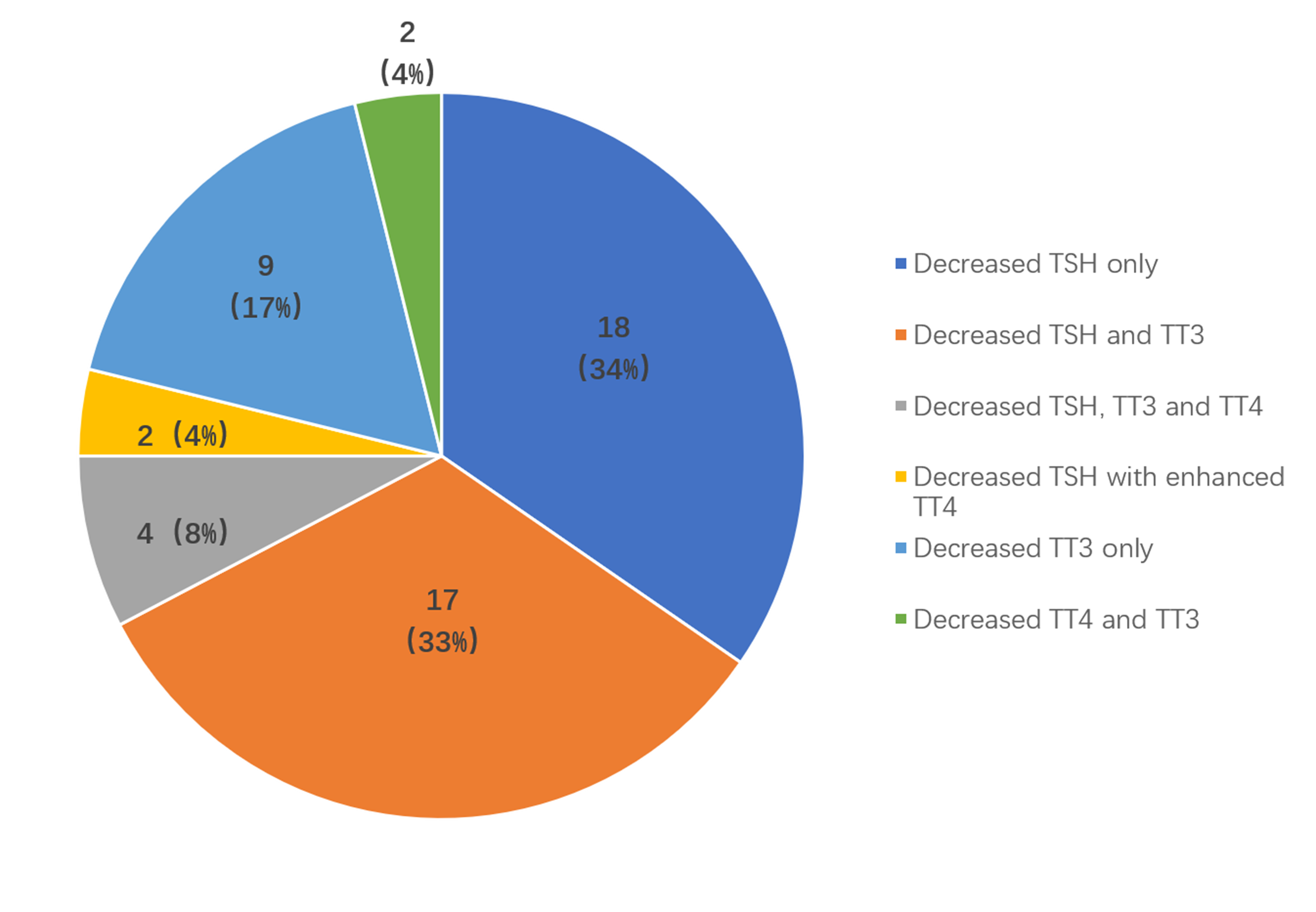

Supplement: Supplementary Figure 1 — The distribution of thyroid hormones abnormalities in COVID-19 patient. [file Image_1.tif]

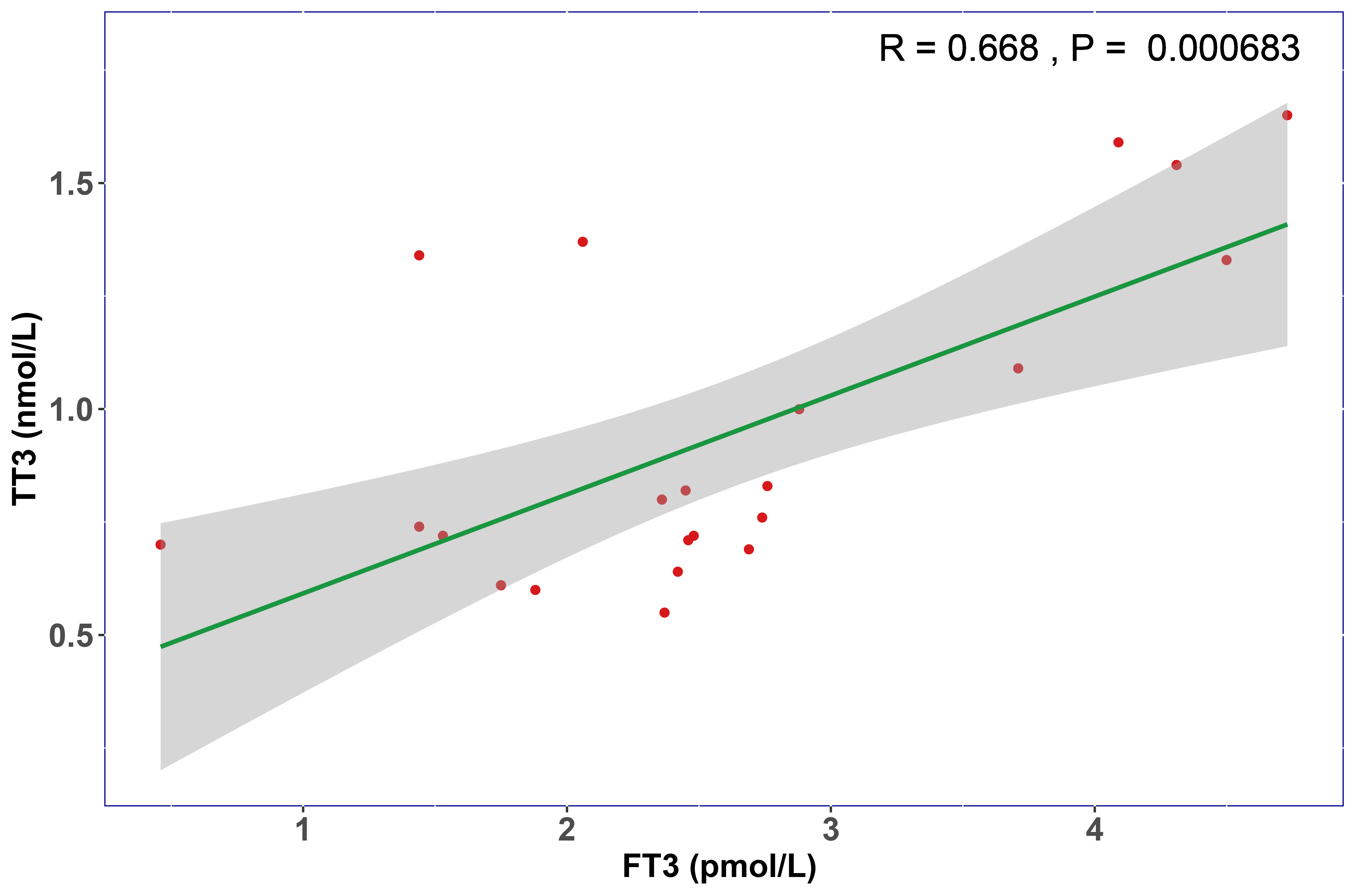

Supplement: Supplementary Figure 2 — The respective association between fT3 and TT3 levels in COVID-19 patients on admission. Correlation analysis shows that fT3 is positively related with TT3 (R = 0.668, P < 0.01). [file Image_2.tif]
