# Supplementary material for: Thyroid Function Abnormalities in COVID-19 Patients
Source: Front Endocrinol (Lausanne). 2021 Feb 19;11:623792. doi: 10.3389/fendo.2020.623792 (PMC7933556; doi:10.3389/fendo.2020.623792)
Supplement: Supplementary file 3 [file DataSheet_1.docx]

| **Table S1** Clinical characteristics and selected laboratory abnormalities of COVID-19 patients with complete or incomplete records of thyroid hormones. | | | |
| --- | --- | --- | --- |
|  | Complete records of thyroid hormones (n=22)  Mean ± SD or n (%) | Incomplete records of thyroid hormones (n=62)  Mean ± SD or n (%) | P value |
| Thyroid function* |  |  |  |
| Dysfunction | 16 (72.7%) | 36 (58.1%) | 0.224 |
| Normal | 6 (27.3%) | 26 (41.9%) |  |
| Clinical classifications on admission |  |  |  |
| Mild and moderate | 3 (13.6%) | 18 (29.0%) | 0.152 |
| Severe and critical | 19 (86.4%) | 44 (71.0%) |  |
| Viral nucleic acid cleaning time (days) | 13.3±7.8 | 12.5±9.1 | 0.722 |
| *Thyroid dysfunction indicates any abnormalities in the levels of TT4, TT3 or TSH. | | | |
